# Digitally enhanced fracture liaison service in Austria—a feasibility analysis

**DOI:** 10.1007/s11657-026-01691-z

**Published:** 2026-03-24

**Authors:** Aaron Pfender, Martina Behanova, Judith Haschka, Johannes Holinka, Daniela Kritsch, Daniel Mattes, Julia Kaufmann, Jochen Zwerina, Roland Kocijan

**Affiliations:** 1 Ludwig Boltzmann Institute of Osteology at Hanusch Hospital of OEGK and AUVA Trauma Center Meidling, 1 st Medical Department Hanusch Hospital, Vienna, Austria; 2 School of Medicine, Metabolic Bone Diseases Unit, Sigmund Freud University Vienna, Vienna, Austria; 3 Department of Orthopaedics and Traumatology, Hanusch Hospital, Vienna, Austria; 4 Mein Gesundheitszentrum Favoriten of OEGK, Vienna, Austria

**Keywords:** Osteoporosis, Fracture, Fracture liaison service, Preventive medicine

## Abstract

***Summary*:**

Fragility fractures often signal untreated osteoporosis. This study shows that a digitally enhanced Fracture Liaison Service (FLS) can identify affected patients in routine hospital care and improve structured assessment and treatment. Dedicated staff remain essential to translate digital solutions into effective fracture prevention.

**Purpose:**

Although Fracture Liaison Services (FLS) are established internationally, structured programs remain scarce in Austria. This study aimed to assess the real-world feasibility of a digitally supported, International Osteoporosis Foundation (IOF)–certified FLS implemented in routine inpatient care.

**Methods:**

We conducted a retrospective, monocentric cohort study at a tertiary care hospital in Vienna, Austria. Hospitalized patients aged ≥ 50 years with major osteoporotic fractures were included in a digital FLS module integrated into the hospital information system between April 2023 and March 2024. Feasibility parameters included patient capture rate, implementation of standardized diagnostics, and initiation or recommendation of osteoporosis therapy.

**Results:**

Overall, 141 patients were enrolled (78% women; mean age 75.8 ± 11.4 years). Vertebral (31.9%) and hip fractures (25.5%) were the most frequent fracture sites. A previous fragility fracture was documented in 46.1% of patients; 24.1% had a prior diagnosis of osteoporosis. In total, 38.1% of all eligible inpatients with osteoporotic fractures were included in the digital FLS. Calcium and vitamin D supplementation was initiated during hospitalization in 73.0% of patients. Specific antiosteoporotic medication was initiated during the inpatient stay in 15.6% and in the outpatient setting in 9.9% and 8.5% of patients, respectively. Treatment was recommended for post-discharge initiation in 56.0% of cases. The most commonly prescribed drugs were denosumab and zoledronic acid.

**Conclusion:**

Implementation of a digitally integrated FLS in routine inpatient care is feasible and enables structured identification, assessment, and treatment recommendation for patients with fragility fractures. However, limited staffing resources and challenges in post-discharge therapy implementation highlight the need for dedicated personnel and improved cross-sectoral care pathways.

**Supplementary Information:**

The online version contains supplementary material available at 10.1007/s11657-026-01691-z.

## Introduction

Over 200 million people worldwide are affected by osteoporosis [1]—a chronic, metabolic skeletal disease characterized by reduced bone quantity and quality, leading to increased fracture risk [2]. Fragility fractures often occur after a low-energy event, such as a simple fall from standing height, and may even arise spontaneously, particularly in individuals over 50 years of age [3]. These fractures predominantly involve the vertebral bodies, the proximal femur, the distal radius, the humerus, and the pelvis—collectively referred to as “major osteoporotic fractures” (MOF) [4]. The incidence of such fractures increases significantly with age, with postmenopausal women being particularly affected [5]. It is estimated that around one in three women and one in five men over the age of 50 will sustain at least one osteoporotic fracture in their lifetime [6].

Of particular importance is the fact that, on average, three out of four fractures (74.2%) in the inpatient setting are associated with osteoporosis, regardless of age or gender [3]. This high proportion makes it clear that these are not rare isolated cases but a central challenge in hospital care that requires systematic recording and treatment.

Fragility fractures are associated with considerable individual limitations including chronic pain, depression, immobility, the need for care, and a significant reduction in quality of life [7]. In the six most populous EU countries (EU6), approximately 2.7 million osteoporotic fractures were recorded in 2017, resulting in a loss of more than one million quality-adjusted life years (QALYs) [8]. In addition to these significant negative socioeconomic impacts, osteoporotic fractures are also a burden on health economics. The associated treatment costs of fragility fractures exceeded the expenditure for other chronic diseases in many European countries [9]. Around 110,000 osteoporotic fractures were registered in Austria in 2019—an alarming increase compared to previous years. In the same year, fracture-related costs accounted for 3.4% of total healthcare expenditure [10]. Compared to 2010 (EUR 799 million) [11], this corresponds to an increase of 501 million euros within 9 years. This puts Austria in sixth place among the EU27 + 2 countries in terms of direct healthcare costs due to fractures [10].

Despite the high clinical and economic relevance, there are serious deficits in secondary prevention. Despite clear guidelines with treatment recommendations after fragility fractures, less than 30% of patients receive antiosteoporotic therapy within 1 year in most countries [12]. In Austria up to 86% of patients with femoral neck fractures remain without antiosteoporotic medication [13]. A previous fracture is one of the highest risk factors for subsequent fractures, especially within the first 2 years (imminent fracture risk) [14, 15].

This is where the structured care approach of the Fracture Liaison Service (FLS) plays a crucial role, an increasingly important international care model [16]. The FLS pursues a multidisciplinary approach for the systematic identification and diagnosis of patients with fragility fractures, and subsequently initiating guideline-compliant treatment and promoting adherence [17]. Numerous international studies have shown that FLS programs contribute to a significant reduction in refracture rates, an increased treatment initiation rate, and improved long-term adherence [18].

Although the FLS concept is established in over 50 countries worldwide, to date structured FLS in Austria are scarce. With the first FLS certified by the International Osteoporosis Foundation (IOF) in Austria, the Hanusch Hospital in Vienna has taken on a pioneering role here [19]. The program provides structured care for patients over 50 years of age with osteoporotic fractures. A special FLS extension within the hospital information system (HIS) enables the multidisciplinary team to rapidly identify potential cases and provides structured documentation. Previous DXA measurements, X-ray images, and laboratory values are automatically retrieved and seamlessly integrated into the FLS database, minimizing manual workload. A digital traffic light system visualizes the treatment status at a glance. All steps of the care process are digitally documented, ensuring transparency, efficiency, optimal workflow and data analysis.

Thus, the goal of the present study was to assess the real-world feasibility of this innovative FLS approach in routine care. Specifically, we evaluated patient capture rates, the implementation of standardized diagnostics and therapy initiation, and potential logistic challenges—providing key insights to support broader adoption of structured secondary fracture prevention across the Austrian healthcare system.

## Methods

### Study design

This retrospective, monocentric cohort study was conducted at the Hanusch Hospital in Vienna, Austria. A structured FLS, certified by the IOF for secondary fracture prevention, was established in 2020, which is organized in close cooperation between the Department of Osteology and the Department of Orthopaedics and Trauma Surgery. The observation period for this study covered the period from April 1, 2023, to March 31, 2024. The conduct of the study was approved by the Ethics Committee of the City of Vienna (EK 22–272-VK).

All hospitalized patients aged 50 years or older who sustained an osteoporotic fracture at a typical site during their hospital stay were included, consistent with an intention to treat approach. Typical sites comprised fractures of the hip, spine, pelvis, proximal humerus, and distal radius. Patients were excluded if they had pathological fractures due to malignancy or polytrauma.

Routine laboratory assessment at admission included extended biochemical screening comprising complete blood count (CBC), electrolytes, creatinine, calcium, and phosphate as part of standard inpatient care. Magnesium was not routinely measured. In addition, 25-OH vitamin D, parathyroid hormone, C-terminal telopeptide of type I collagen, and osteocalcin were obtained on the following day. C-terminal telopeptide of type I collagen, alkaline phosphatase, creatinine, estimated glomerular filtration rate, osteocalcin, 25-hydroxyvitamin D, phosphate, and calcium were automatically integrated into the FLS interface to streamline and optimize the workflow of the FLS team. The FLS team was automatically notified via the hospital information system (HIS). The FLS coordinator then assessed the patients in person, conducting a detailed medical history directly at the patient’s bedside and collecting relevant data using a tablet-based case report form (medical history, risk of falls, previous fractures, comorbidities, and medication) [19]. All data were collected and stored electronically using a pre-programmed questionnaire. The therapeutic decision was subsequently made by the physicians of the FLS team based on national and international guidelines. Diagnostic considerations and treatment decisions were then summarized in a written report and documented in the patient’s discharge letter. Previous DXA measurements, X-ray images, and laboratory values are automatically integrated into the interface and the FLS database. A digital traffic light system showed the treatment status: red for new cases, yellow after initial contact by nursing, and green after medical decision.

### Data collection

Data were collected as part of the clinical FLS workflow by specially trained nursing staff (FLS nurses), who completed standardized Case Report Forms (CRF) at the bedside. Age and gender were automatically transferred from the HIS to the CRF. All other information was either collected directly using the structured interview or taken from the electronic patient file. Prior to analysis, all data sets were pseudonymized and stored on a password-protected server at the Ludwig Boltzmann Institute of Osteology; access was restricted to authorized project staff.

The following demographic and lifestyle-related variables were recorded: height, weight, body mass index (BMI), residence (nursing home), primary care provider, acute and previous fractures, family history of hip fractures, age at menopause (in women), alcohol (> 3 units per day) and tobacco consumption (> 5 cigarettes per day). In addition, the FLS nurses recorded mobility, and social factors, including the degree of mobility, any wheelchair dependency, falls within the last 12 months, housing situation, dental health, and diet (balanced, vegetarian, and vegan).

Fracture history was assessed using separate binary (yes/no) variables for each fracture site.

The fracture documentation included fracture sites and categorized MOF and “other fractures.” The analysis was divided into superordinate categories (upper extremity, lower extremity, ribs, sternum, cervical region); fractures distal to the hip were assigned to the lower extremity, while radius fractures were recorded separately. Earlier fractures were additionally classified according to accident mechanism (atraumatic, fall, high-energy trauma).

Comorbidities and medications were systematically recorded. Endocrinological, rheumatological, gastrointestinal, neurological, psychiatric, and oncological diagnoses were documented; tumour diseases were reported as active or past, rare entities such as uterine carcinoma, acute lymphoblastic leukaemia (ALL), tongue, liver, renal cell carcinoma (RCC), and thyroid carcinoma (SD-CA) were analyzed as separate categories. In parallel, any medication with a potential impact on bone health or fall risk (including glucocorticoids, proton pump inhibitors, calcium, and vitamin D supplements) was recorded and added to the dataset.

The DXA status was recorded in a structured manner and categorized as “performed,” “externally available,” “ordered,” “refused,” or “not required.” If T-scores were available for the lumbar spine, hip, or femoral neck, these were automatically transferred from the HIS to the CRF, as was the trabecular bone score (TBS) based on image analysis of the lumbar spine in the L1–L4 region. The FRAX algorithm of the University of Sheffield was used for the standardized estimation of the individual 10-year fracture risk. As type 2 diabetes mellitus is not considered an independent risk factor in the FRAX model, the variable “rheumatoid arthritis” was used as a surrogate parameter, in line with the evidence on the equivalence of fracture risk [20]. The FRAX fracture risk was calculated for each patient including and excluding recent index fracture event.

At the time of the index fracture, calcium, vitamin D supplements, as well as specific antiosteoporotic medication (AOM) were recorded. Corresponding information was also collected for the treatment recommendations made as part of the FLS. Unclear or missing information was stated as “unknown.”

The planned implementation of the therapy was differentiated depending on the intended treatment setting: initiation during the inpatient stay, initiation within the FLS outpatient clinic (within 8–12 weeks after discharge), or extramural implementation, for example by the general practitioner or specialist. In cases where none of these categories applied, there was the option to provide a free response under “Other.” Any reasons for not initiating treatment—such as calcium metabolism disorders, secondary hyperparathyroidism, a recent fracture or a reduced general condition—were recorded using predefined categories or added via a free text field.

For the final evaluation, the HIS was used to individually check whether the recommended drug therapy was actually implemented and in which institution; each documented implementation was classified accordingly as “calcium and vitamin D substitution” or “specific therapy.”

### Statistical analysis

We summarized baseline characteristics with appropriate descriptive statistics: continuous variables as mean and standard deviation or median and interquartile range (for non-normally distributed data) were calculated. Categorical variables were reported as absolute and relative frequencies.

Hypothesis tests and *p*-values were not used, as no formal group comparison was planned.

A capture rate analysis was performed to evaluate FLS using as denominator the number of inpatients admitted to Orthopaedics/Trauma with prespecified fracture diagnoses over the same period, obtained from the controlling department of the Hanusch hospital. Capture Rate (%) = (number enrolled in FLS/total eligible fracture inpatients) × 100. All results are reported as mean ± SD or median (IQR) if not stated otherwise.

## Results

### Patient characteristics and fracture profile

During the 12-month observation period, a total of 141 patients (78% females) were enrolled in the FLS program at Hanusch Hospital. 53.9% were contacted by the FLS nurse during their hospital stay. The mean age was 75.8 years (SD ± 11.4), ranging from 51 to 98 years. The median BMI was 24 kg/m^2^ (IQR 21.5–29.0). A detailed BMI classification showed that 5.7% of patients were underweight, 43.3% had normal weight, 22.0% were overweight, and 20.6% were obese (in 8.5% of patients, BMI status was not recorded). Most patients (80.1%) reported a balanced diet. An analysis of comorbidities showed that type 2 diabetes and thyroid disorders were the most frequently diagnosed conditions (13.5% each, see Sppl. Tbl. 1).

Relevant summary of the demographic data is summarized in Table 1 and Sppl. Tbl. 1.

**Table 1:** Demographic data. Acronyms: median (interquartile range, IQR) and mean (standard deviation, SD)

Demographic data		
Category	Value	
Total number of patients	141	
Age (mean, SD)	75.8 (11.4)	
Sex, *N* (%)		
Female	110 (78.0)	
Male	31 (22.0)	
FLS contact, *N* (%)		
Patients contacted within their hospital stay	76 (53.9)	
Patient contacted after being released from the hospital	53 (37.6)	
No data about time	12 (8.5)	
History of previous fracture		
Yes	65 (46.1)	
No	76 (53.9)	
Physical & nutrition data		
BMI (median, IQR)	24.0 (21.5 29.0)	
Recommended therapy		
No AOM	4 (2.8)	
Denosumab	25 (17.7)	
Zoledronate	19 (13.5)	
Teriparatide	8 (5.7)	
Romosozumab	1 (0.7)	
Other therapy	9 (6.4)	
Only calcium + vitamin D	32 (22.7)	
Unknown (missing data)	43 (30.5)	
Current therapy at the time of Fx		
No therapy	64 (45.4)	
Teriparatide	1 (0.7)	
Calcium + vitamin D	26 (18.4)	
Alendronate	2 (1.4)	
Denosumab	6 (4.3)	
Risendronate	1 (0.7)	
Ibandronate	3 (2.1)	
Unknown	38 (27.0)	
**DXA results (*T*-scores)**	***N***	**Value**
Lumbal Spine	15	−2.0 (SD 1.3)
Hip	12	−2.0 (SD 0.8)
Femoral neck	15	−2.2 (SD 1.4)
Trabecular Bone Score	13	−1.26 (SD 0.14)
**Laboratory results**		
**Parameter, unit**	***N***	**Value**
Vitamin D, nmol/l, median (IQR)	88	76.0 (IQR 47.3–103.5) [Ref: 75–250]
Beta-crosslaps, nmol/l	86	0.55 (IQR 0.36–0.76) [Ref: 0.18–1.06]
Alkaline phosphate, U/l	99	90.0 (IQR 68.0–125.0) [Ref: 30–120]
Creatinine, mg/dl	100	0.77 (IQR 0.64–0.97) [Ref: 0.51–0.95]
GFR/1.7m2KO(CKDEPI), ml/min/1.73m^2^	100	77.5 (IQR 59.5–88.8) [Ref: 90–200]
Osteocalcin, ng/ml	86	16.5 (IQR 11.7–21.0) [Ref: 6.5–42.3]
Phosphate, mmol/l (*N *= 89)	89	0.98 (IQR 0.85–1.11) [Ref: 0.81–1.45]
Calcium, mmol/l (*N *= 104)	104	2.26 (SD 0.12) [2.20–2.65]

**Fig. 1:**
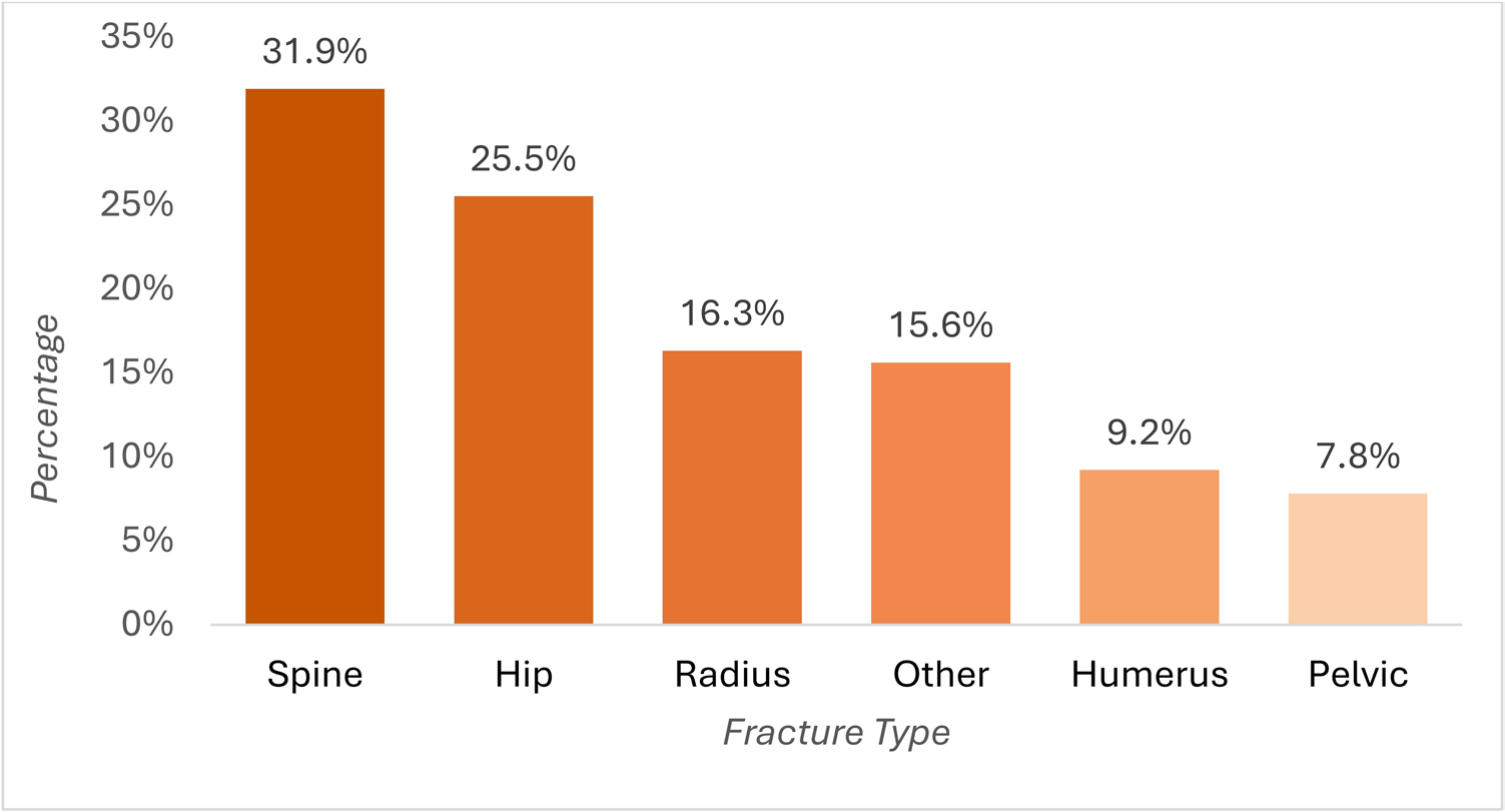
Fracture sites present at hospital admission. Bars represent the percentage of patients with fractures at the specified anatomical sites at the time of admission. Patients could present with fractures at more than one site; therefore, categories are not mutually exclusive and percentages do not sum to 100%

Among the FLS patients, the most common fracture types were vertebral fractures (31.9%, *n *= 45), followed by hip (25.5%, *n *= 36), radius (16.3%, *n *= 23), humerus (9.2%, *n *= 13), and pelvic fractures (7.8%, *n *= 1) (Fig. 1). Other fracture sites were documented in 15.6% (*n *= 22) of patients, including fractures of the lower extremities (8.5%, *n *= 12), upper extremities and dens (1.4% each, *n *= 2), ribs (2.1%, *n *= 3), and sternum (0.7%, *n *= 1). The trauma impact was documented in 83% (*n *= 117) of cases, with 72.3% (*n *= 102) attributed to low traumatic events.

**Fig. 2:**
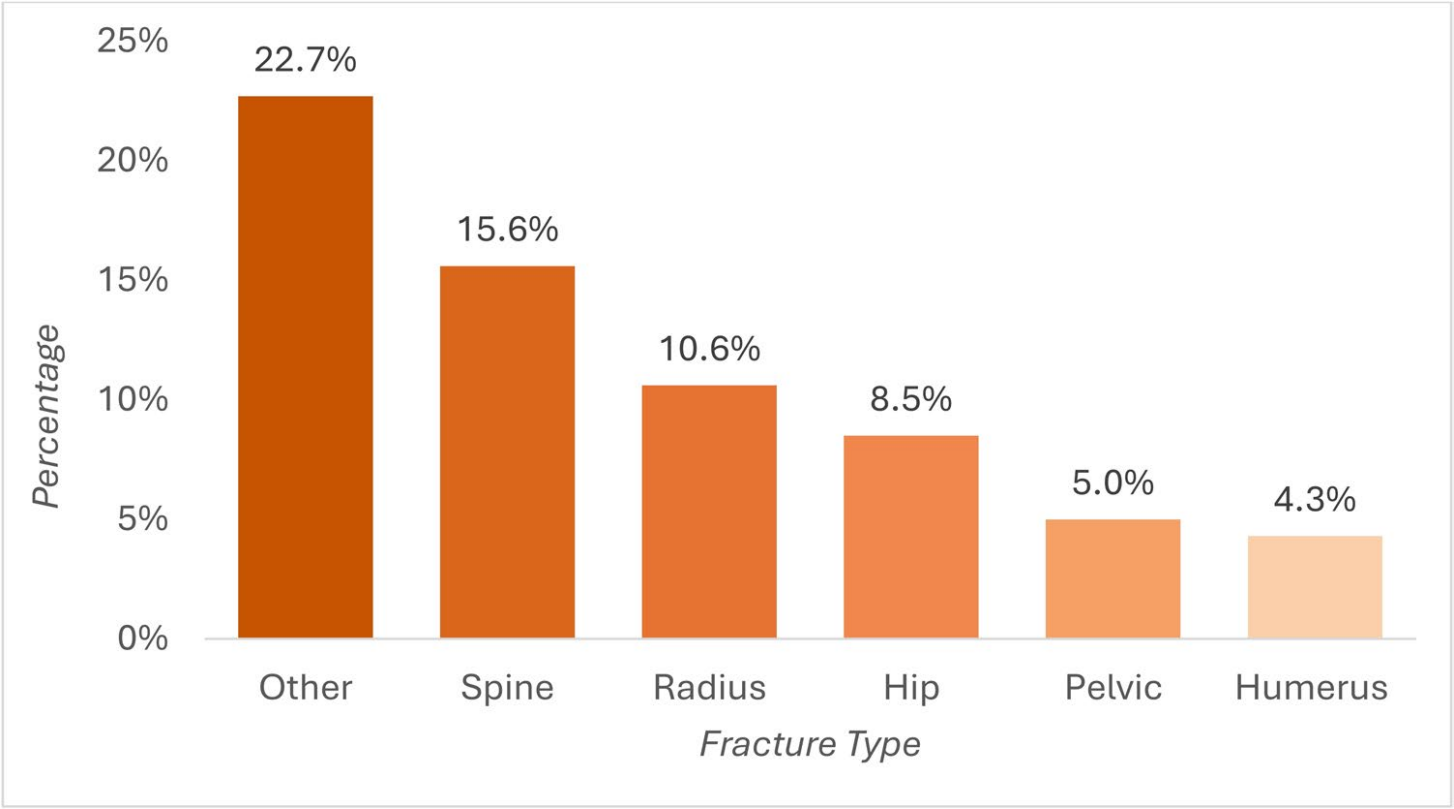
Percentage of patients with a history of fracture at each site. Bars represent the percentage of patients reporting a prior fracture at each anatomical site. Patients could report more than one fracture type; therefore, categories are not mutually exclusive and percentages do not sum to 100%

A history of previous fracture was reported in 46.1% (*n *= 65) of patients, consisting of vertebral fractures in 15.6% (*n *= 22), radius fractures in 10.6% (*n *= 15), hip fractures in 8.5% (*n *= 12), pelvic fractures in 5.0% (*n *= 7), and humerus fractures in 4.3% (*n *= 6) (Fig. 2). Previous fractures in other fracture locations were found in 22.7% (*n *= 32), including fractures of lower extremities (*n *= 17), ribs (*n *= 6), upper extremities, cervical spine, clavicle, and skull (*n *= 1 each).

### Fracture risk

**Fig. 3:**
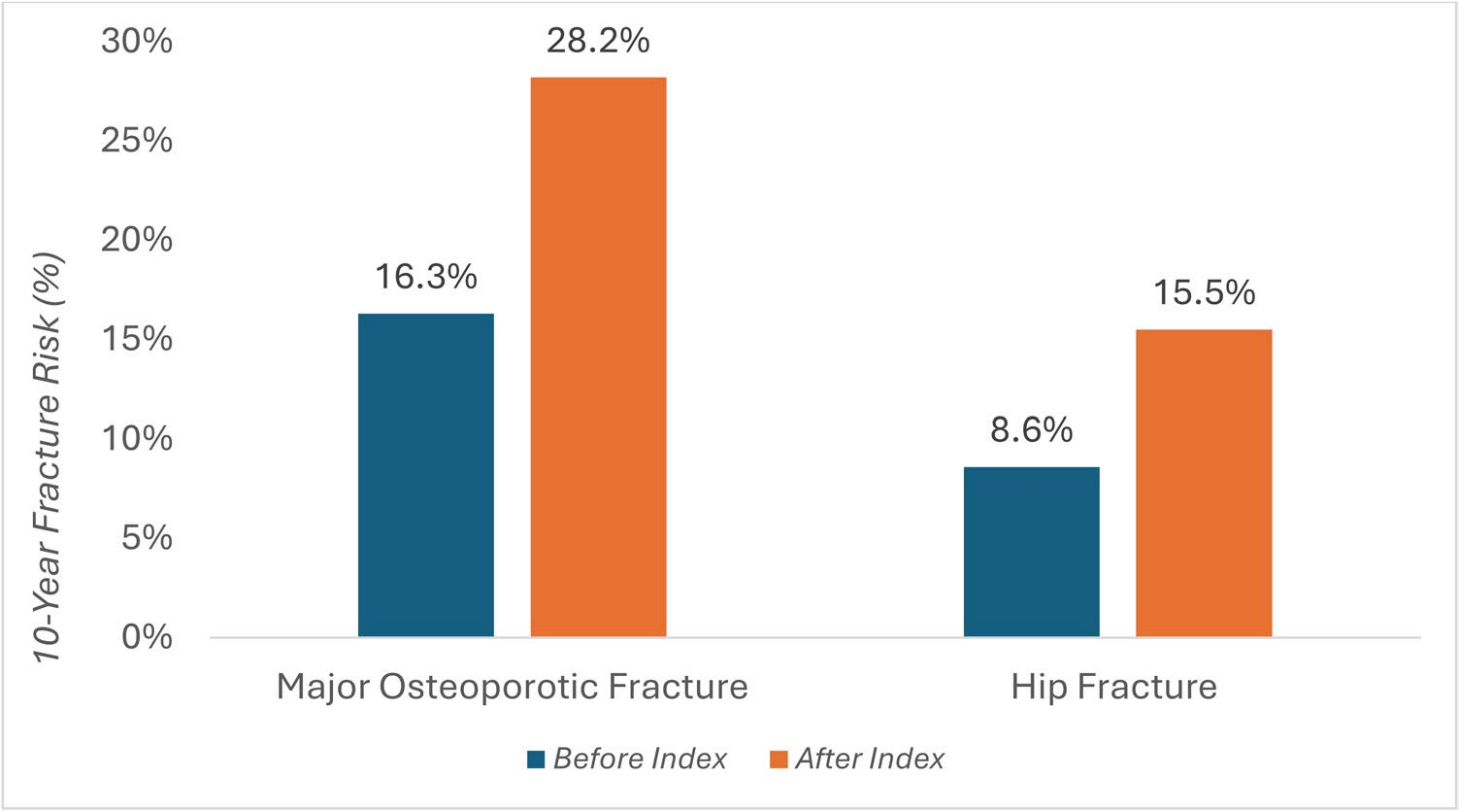
Ten-year fracture probability before and after index fracture. The bar chart shows the 10-year probability of a major osteoporotic fracture (MOF) and a hip fracture, calculated by FRAX, both before (blue bars) and after the index fracture (orange bar) in patients included in the Fracture Liaison Service (FLS)

Before the index fracture occurred, 24.1% (*n *= 34) of patients had already been diagnosed with osteoporosis. In addition, FRAX assessments indicated a consistently high 10-year fracture risk both before and especially after the index event (Fig. 3). Risk for MOF and hip fractures was 28.2 ± 13.5% and 15.5 ± 11.6%, respectively, at the time of hospital admission. The FRAX was also calculated without the current fracture and yielded values of 16.3 ± 9.9% for MOF and 12.6 ± 7.6%, respectively.

### Antiosteoporotic treatment: before and after index fracture

Among the 141 patients included, 18.4% (*n *= 26) were receiving calcium and vitamin D substitution, 9.2% (*n *= 13) were treated with specific AOM, and 45.4% (*n *= 64) were not receiving any anti-osteoporosis medication (AOM) at the time of fracture. Information on prior AOM use was unavailable for 38 patients (27%).

Among the 65 FLS patients with a history of a previous fracture, 33 patients (50.8%) were not receiving any AOM at the time of the index fracture. Fourteen patients (21.5%) were receiving calcium and vitamin D substitution, and nine (13.8%) were treated with specific AOM (Table 1).

### Treatment initiation and administration

**Fig. 4:**
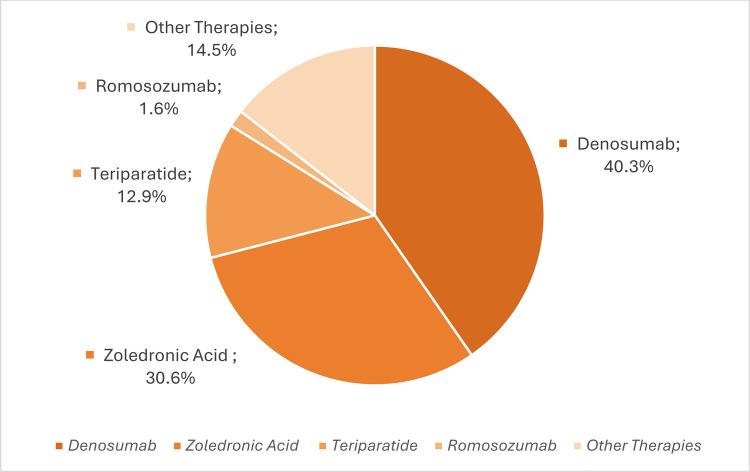
Distribution of recommended AOM treatment among patients with specific AOM recommendation (*n *= 62). Percentages refer to proportions within this subgroup

Within the FLS framework, no AOM was recommended for 4 patients (2.8% of the total cohort). Calcium and vitamin D substitution alone was recommended in 22.7% (*n *= 32). Specific AOM was recommended in 44.0% (*n *= 62). Denosumab was the most frequently recommended agent (*n *= 25, 40.3%), followed by zoledronic acid (*n *= 19, 30.6%). Osteo-anabolic agents were recommended less frequently, with teriparatide (*n *= 8) and romosozumab (*n *= 1) in 12.9% and 1.6% of cases, respectively (Fig. 4).

In 73% of patients (*n *= 103), calcium and vitamin D substitution was already initiated during their inpatient stay. Specific AOM was initiated or recommended within the FLS cohort in different healthcare settings. In 15.6% of cases (*n *= 22), AOM was actually administered in an inpatient setting and 9.9% of patients (*n *= 14) received therapy at an outpatient healthcare center. Moreover, AOM was administered in 8.5% of cases in the outpatient unit of the hospital. In 56% of cases (*n *= 79), initiation of specific anti-osteoporotic therapy was recommended after discharge from the hospital in an outpatient setting or at the family doctor. However, it was not possible to verify whether the recommended treatment was initiated as recommended. In 8.5% of cases (*n *= 12), there was no documentation of AOM administration or recommendation.

Reasons for postponing the start of anti-osteoporotic therapy comprised most frequently vitamin D insufficiency (*n *= 35), calcium metabolism disorders (*n *= 18), and secondary hyperparathyroidism (*n *= 7). In 13 cases, treatment was postponed due to a recent refracture. In one case, a significantly reduced general condition in the context of sepsis led to the decision against immediate specific treatment.

### Capture rate of the FLS

During the selected 1-year survey period, a total of 307 patients were admitted to Hanusch Hospital with a typical osteoporotic fracture site. Of these, 117 cases—corresponding to 38.1%—were identified and recorded by the digital FLS. A detailed breakdown of the case numbers and percentage distributions by diagnosis can be found in Table 2.

**Table 2:** Fracture liaison service capture rates by fracture diagnosis during 1-year study period

Diagnose (ICD-10)	Number of in-patients admitted to the hospital (*N*)	Number of in-patients captured by FLS (*N*)	FLS—capture rate (%)
Pelvic and lumbar spine (S-32)	74	45	60.8
Shoulder and upper arm (S-42)	36	13	36.1
Forearm (S-52)	84	23	27.4
Femur and hip (S-72)	113	36	31.9
**Total**	**307**	**117**	**38.1**

## Discussion

This study describes the implementation of a digitally supported Fracture Liaison Service (FLS) within the Austrian healthcare system. The digital integration of structured workflows, real-time data retrieval, and standardized documentation within an existing hospital information system enabled systematic case identification and management of patients with fragility fractures. Our findings demonstrate that a structured, interdisciplinary approach is feasible in a clinical routine setting and that it improves the diagnostic and therapeutic quality of secondary fracture prevention.

Vertebral fractures and hip fractures were the predominant localizations in our FLS. Both fracture types are of particular clinical and socioeconomic relevance due to their association with high morbidity, functional decline, and increased mortality. In Austria alone, hip fractures account for an estimated annual direct cost burden exceeding 70 million euros [21], emphasizing the urgency of structured prevention programs. The high proportion of vertebral fractures in our cohort exceeds the rates typically reported in other FLS cohorts (approximately 10–15%) [22, 23]. This likely reflects improved inpatient identification, particularly because a substantial proportion of our inpatients were referred directly from our dedicated spine outpatient clinic. At the same time, it underlines a persistent diagnostic gap, as the majority of vertebral deformities remain undetected in routine radiology reports. Given the prognostic significance of vertebral fractures as strong predictors of subsequent fractures, their early recognition is essential. The integration of artificial intelligence (AI) algorithms for opportunistic vertebral fracture detection in routine imaging may therefore represent an important next step to expand the diagnostic reach of FLS programs [24].

A capture rate of 38.1% was achieved during the 12-month observation period, which is within the lower range of international benchmarks but still reflects good feasibility in a newly implemented national program. However, this result must be interpreted with appropriate self-criticism. Our FLS operated exclusively within the inpatient setting and lacked continuous staffing, as the position of a dedicated FLS nurse could not be maintained throughout the entire study period. Without an FLS nurse, and with only minimal additional staff capacity, systematic case finding cannot be performed reliably, even when digital tools are available. A once-weekly FLS screening is clearly insufficient to ensure adequate patient capture. These structural constraints represent the main reason for the comparatively high number of missed eligible patients.

In addition, even among the identified and enrolled patients, only about 50% were seen in person on the ward. The remaining patients were contacted through telemedicine. While this hybrid approach is a legitimate and increasingly relevant component of modern FLS structures, it also requires dedicated staff resources as well as suitable hardware and software solutions. Telephone or video-based counselling, digital transfer of lab requests, structured electronic documentation, and follow-up coordination cannot be delivered sustainably without dedicated FLS-time and adequate technical infrastructure. Therefore, any future expansion of FLS services must ensure stable staffing with a dedicated FLS nurse, continuous high-frequency screening, and investment in secure, user-friendly telemedical systems to support a scalable and cross-sectoral care model [19, 25].

Consistent with previous international reports, our data confirm substantial diagnostic and therapeutic deficits in osteoporosis care prior to FLS intervention. Only 10.6% of patients had undergone a recent DXA measurement, and fewer than one in four had a documented osteoporosis diagnosis despite high calculated FRAX probabilities for major osteoporotic and hip fractures—well above the treatment thresholds defined by national guidelines. These findings illustrate a major missed opportunity for primary and secondary prevention. Nearly half of the patients had already experienced a previous fragility fracture, underscoring the concept of “imminent fracture risk” in case of vertebral fractures or hip fractures and the need for earlier intervention [20].

The FLS intervention markedly improved the initiation of osteoporosis therapy. Following structured evaluation, 73% of patients received calcium and vitamin D supplementation, while specific anti-osteoporotic medication was recommended for 44% of patients. These results correspond to international best-practice standards, which define a therapy initiation rate above 40% as indicative of a high-quality FLS [26]. Before FLS contact, only 9.2% (*N *= 13) of patients had been treated with anti-osteoporotic agents, reflecting the well-known therapeutic gap after fragility fractures [6, 27]. The observed increase following structured FLS involvement highlights the effectiveness of coordinated, multidisciplinary care in addressing this unmet need.

Although inpatient initiation of calcium and vitamin D substitution was highly feasible, the initiation of specific anti-osteoporotic medication remained limited to 15.6% of patients during hospitalization. In many cases, immediate therapy was postponed due to transient contraindications such as vitamin D deficiency, calcium imbalance, or acute intercurrent illness. For these patients, treatment was typically scheduled within 8–12 weeks after discharge in the FLS outpatient clinic. This model of distributed care reflects the necessary collaboration between inpatient and outpatient sectors but also exposes potential coordination gaps. For more than half of the cases, it was not possible to verify whether the recommended therapy had been implemented after discharge. Issuing such evidence-based treatment recommendations represents the minimum standard of an FLS and ensures that patients are appropriately guided toward secondary fracture prevention. This highlights a major challenge for the long-term success of FLS programs, ensuring continuity of care and adherence across healthcare sectors. The establishment of digital feedback systems, national FLS registries, and automated follow-up alerts could substantially improve communication and outcome tracking.

Denosumab was the most frequently prescribed specific agent in our cohort, which is in line with other FLS centers [28]. Denosumab demonstrates a robust antifracture efficacy across all major fracture sites in both women and men, which further supports their widespread use in routine FLS care. The second most commonly administered drug was zoledronic acid. The inpatient administration of zoledronic acid is well tolerated and safe in hip fracture patients [29] and was reported to decrease mortality, even after a single dose. Zoledronic acid also reduced the vertebral fracture incidence, however not non-vertebral or hip fractures in previous studies [30]. The preference of DMAB and zoledronic acid also reflects the characteristics of our population, consisting predominantly of older, multimorbid patients. Potent, well-tolerated antiresorptive therapy is often the most pragmatic option, even if osteoanabolic therapy would bring greater benefits [31]. The relatively low proportion of osteoanabolic therapy must also be interpreted in the context of national reimbursement policies. During the study period (April 2023–March 2024), romosozumab was not yet included in the Austrian reimbursement catalogue. Teriparatide was available but subject to strict approval procedures, and its requirement for daily self-administration represented an additional barrier for many patients—particularly those of advanced age or with functional limitations. Thus, the limited use of anabolic agents in our cohort reflects structural and practical access barriers rather than a lack of clinical indication. With the recent reimbursement of romosozumab in Austria and growing evidence supporting sequential anabolic–antiresorptive strategies, an expansion of osteoanabolic prescribing can be expected in future FLS implementations.

One of the major strengths of this project is the direct integration of the FLS into the hospital information system with automated case finding, enabling FLS activities to be conducted within existing clinical workflows rather than through separate platforms. The FLS at Hanusch Hospital was implemented as an integrated IT module within the hospital information system, supporting staff-based case identification, structured digital data entry, and real-time visualization of patient status via a traffic-light interface. This streamlined workflow reduced manual effort, improved data quality, and enabled efficient documentation and follow-up.

From a methodological perspective, the digital structure also created a solid data infrastructure for scientific evaluation, facilitating harmonization, benchmarking, and quality control. The system has since been extended to the affiliated outpatient health centers, which now use the same platform for follow-up visits—ensuring continuity of care and supporting the development of an interconnected FLS network.

The study has several limitations. Its retrospective, single-center design limits the generalizability of findings, and the absence of a control group precludes causal inference regarding fracture reduction or adherence outcomes. Data on refracture rates, mortality, and long-term therapy persistence were not available. Furthermore, therapy implementation outside the hospital could not be verified in all cases, and economic parameters were not systematically recorded. In line with current guidelines, therapy initiation was not postponed for additional imaging in patients with a clear indication for treatment. However, in patients with peripheral fragility fractures, DXA and spine radiographs were systematically recommended in the outpatient setting to refine risk stratification and support individualized long-term treatment decisions. Furthermore, as our FLS operated exclusively within the inpatient setting during the observation period, patients managed solely in the emergency department or in outpatient care were not systematically identified. This structural limitation may have led to an underrepresentation of the overall fragility fracture population and highlights the need to expand FLS strategies beyond the inpatient setting to ensure comprehensive secondary fracture prevention. Despite these constraints, the results provide a strong proof-of-concept foundation for future prospective and multi-centre research.

## Conclusion

This study demonstrates that a digitally supported, multidisciplinary Fracture Liaison Service is both feasible and effective. The program improved the identification and management of osteoporosis after fragility fractures and offers a scalable framework for secondary fracture prevention. However, the findings clearly show that digital tools alone are insufficient. Dedicated human resources—particularly continuous staffing and a trained FLS nurse—are essential to ensure reliable case finding, patient counselling, and therapeutic implementation. Further optimization is needed, especially regarding capture rates, cross-sectoral integration, and broader access to osteoanabolic therapies. With these refinements, this combined human–digital model provides a strong foundation for a sustainable national strategy to reduce fracture burden and healthcare costs in aging populations.

## Data Availability

Data supporting this publication can be accessed at our institutional digital data repository for published research via creed.lbg.ac.at.

## Appendix

Below is the link to the electronic supplementary material.
Supplementary Material 1 (DOCX 24.4 KB)
