# Supplementary material for: Digitally enhanced fracture liaison service in Austria—a feasibility analysis
Source: Arch Osteoporos. 2026 Mar 24;21(1):55. doi: 10.1007/s11657-026-01691-z (PMC13013220; doi:10.1007/s11657-026-01691-z)
Supplement: Supplementary file 1 — Supplementary Material 1 (DOCX 24.4 KB) [file 11657_2026_1691_MOESM1_ESM.docx]

| Category | Value | |  | % |
| --- | --- | --- | --- | --- |
| Nutrition, N (%) |  |  | |  |
| Balanced diet | 113 | |  | 80.1% |
| Vegetarian diet | 1 | |  | 0.1% |
| Vegan diet | 1 | |  | 0.1% |
| Unknown | 26 (18.4) |  | |  |
| MEDICAL CARE DATA |  | |  |  |
| Living in nursing home |  |  | |  |
| yes | 6 (4.3) | |  | 4.3% |
| no | 135 (95.7) |  | |  |
| Contact information about patient’s’ general practitioner |  |  | |  |
| Available | 33 (23.4) |  | |  |
| Not known | 108 (76.6) |  | |  |
| Dental status N (%) |  |  | |  |
| Normal dental condition | 54 (38.3) | |  |  |
| Restored dentition | 38 (27.0) | |  |  |
| Unclear | 10 (7.1) |  | |  |
| Under treatment | 2 (1.4) |  | |  |
| Treatment indicated | 6 (4.3) |  | |  |
| Unknown | 31 (22.0) |  | |  |
|  |  |  | |  |
| GENERAL RISK FACTORS |  | |  |  |
| Mobility |  |  | |  |
| Unresricted mobility | 70 (49.6) | |  | 49.6% |
| Walking aid | 37 (26.2) | |  | 26.2% |
| Wheelchair use | 2 (1.4) | |  | 1.4% |
| Unknown | 32 (22.7) |  | |  |
| Risk of falling |  |  | |  |
| No risk | 74 (52.5) | |  | 52.5% |
| 1-2/Year | 22 (15.6) | |  | 15.6% |
| Frequent risk | 16 (11.3) | |  | 11.3% |
| Missing data | 29 (20.6) |  | |  |
| Parental history of osteoporosis |  |  | |  |
| Maternal osteoporosis diagnosis | 12 (8.5) | |  | 8.5% |
| Paternal osteoporosis diagnosis | 5 (3.5) | |  | 3.5% |
| Osteoporosis in both parents | 1 (0.7) | |  | 0.7% |
| Unknown | 123 (87.2) |  | |  |
|  |  |  | |  |
| Hysterectomy | 12 | |  | 10.9% |
| Ovariectomy | 2 | |  | 1.8% |
| ACUTE DISEASE |  | |  |  |
| Thyroid disease | 19 | |  | 13.5% |
| Diabetes mellitus | 19 | |  | 13.5% |
| Depression | 13 | |  | 9.2% |
| Asthma/COPD | 12 | |  | 8.5% |
| Chronic renal insuff. | 10 | |  | 7.1% |
| Acute malignancies | 7 | |  | 5.0% |
| Gastritis/Reflux | 6 | |  | 4.3% |
| Rheumatoid arthritis | 5 | |  | 3.5% |
| Parkinson's disease | 5 | |  | 3.5% |
| Celiac desease | 3 | |  | 2.1% |
| Liver disease | 2 | |  | 1.4% |
| Epilepsy | 2 | |  | 1.4% |
| HISTORY OF MALIGNANCIES |  | |  |  |
| History of cancer | 14 | |  | 9.9% |
| Breast carcinoma | 8 | |  | 5.7% |
| Urothelial carcinoma | 2 | |  | 1.4% |
| Melanoma | 1 | |  | 0.7% |
| Bronchial carcinoma | 1 | |  | 0.7% |
| Colon carcinoma | 1 | |  | 0.7% |
| Prostate carcinoma,  Non-Hodgkins lymphoma,  Plasmacytoma | 0 | |  | 0% |
| Other malignancies | 7 | |  | 5% |
| Thyroid carcinoma | 1 | |  | 0.7% |
| Uterine carcinoma | 1 | |  | 0.7% |
| Acute lymphoblastic leukaemia | 1 | |  | 0.7% |
| Lung carcinoma | 1 | |  | 0.7% |
| Liver carcinoma | 1 | |  | 0.7% |
| Renal cell carcinoma | 1 | |  | 0.7% |
| Prior radiation therapy | 8 | |  | 5.7% |
| GENERAL MEDICATION |  | |  |  |
| Proton pump inhibitors (PPI) | 28 | |  | 19.9% |
| Antidepressants | 7 | |  | 5.0% |
| Glucocorticoids | 6 | |  | 4.3% |
| Anti-hormone therapy | 1 | |  | 0.7% |
| Aromatase inhibitors 0 (0%) | 0 | |  | 0% |
| DXA recommendation |  | |  |  |
| Ordered | 2 | |  | 1.4% |
| Performed in the last 24 months | 15 | |  | 10.6% |
| Recommended | 20 | |  | 14.2% |
| Not necessary | 28 | |  | 19.9% |
| Performed externally | 25 | |  | 17.7% |
| Unknown | 51 (36.2) | |  |  |

Supplementary Table 1: Full Demographic Dataset: Acronyms: median (Interquartile range, IQR), mean (Standard deviation, SD)
